# Supplementary material for: Increased frequency of repeat expansion mutations across different populations
Source: Nat Med. 2024 Oct 1;30(11):3357–68. doi: 10.1038/s41591-024-03190-5 (PMC11564083; doi:10.1038/s41591-024-03190-5)
Supplement: Supplementary file 1 — Reporting Summary [file 41591_2024_3190_MOESM1_ESM.pdf]

nature portfolio

Corresponding author(s): Arianna Tucci

Last updated by author(s): 04/07/2024

Reporting Summary

Nature Portfolio wishes to improve the reproducibility of the work that we publish. This form provides structure for consistency and transparency in reporting. For further information on Nature Portfolio policies, see our Editorial Policies and the Editorial Policy Checklist.

Statistics

For all statistical analyses, confirm that the following items are present in the figure legend, table legend, main text, or Methods section.

n/a	Confirmed	
☐	☒	The exact sample size (*n*) for each experimental group/condition, given as a discrete number and unit of measurement
☒	☐	A statement on whether measurements were taken from distinct samples or whether the same sample was measured repeatedly
☒	☐	The statistical test(s) used AND whether they are one- or two-sided<br>*Only common tests should be described solely by name; describe more complex techniques in the Methods section.*
☒	☐	A description of all covariates tested
☒	☐	A description of any assumptions or corrections, such as tests of normality and adjustment for multiple comparisons
☐	☒	A full description of the statistical parameters including central tendency (e.g. means) or other basic estimates (e.g. regression coefficient) AND variation (e.g. standard deviation) or associated estimates of uncertainty (e.g. confidence intervals)
☐	☒	For null hypothesis testing, the test statistic (e.g. *F*, *t*, *r*) with confidence intervals, effect sizes, degrees of freedom and *P* value noted<br>*Give P values as exact values whenever suitable.*
☒	☐	For Bayesian analysis, information on the choice of priors and Markov chain Monte Carlo settings
☒	☐	For hierarchical and complex designs, identification of the appropriate level for tests and full reporting of outcomes
☐	☒	Estimates of effect sizes (e.g. Cohen's *d*, Pearson's *r*), indicating how they were calculated

Our web collection on statistics for biologists contains articles on many of the points above.

Software and code

Policy information about availability of computer code

Data collection	https://github.com/Illumina/ExpansionHunter (EHv322)<br>https://github.com/Illumina/REViewer (one version available)
Data analysis	https://github.com/bharatij/ExpansionHunter_Classifier (one version available)<br>https://odelaneau.github.io/shapeit4/<br>https://github.com/slowkoni/rfmix (version 2)<br>http://faculty.washington.edu/browning/beagle/beagle.html (version 5.4)<br>https://github.com/chrisclarkson/gel/tree/main/HTT_work<br>https://github.com/Illumina/gvcfgenotyper<br>https://github.com/nam10/C9_Penetrance

For manuscripts utilizing custom algorithms or software that are central to the research but not yet described in published literature, software must be made available to editors and reviewers. We strongly encourage code deposition in a community repository (e.g. GitHub). See the Nature Portfolio guidelines for submitting code & software for further information.

Data

Policy information about availability of data

All manuscripts must include a data availability statement. This statement should provide the following information, where applicable:

- Accession codes, unique identifiers, or web links for publicly available datasets
- A description of any restrictions on data availability
- For clinical datasets or third party data, please ensure that the statement adheres to our policy

For the 100K GP, full data is available in the Genomics England Secure Research Environment. Access is controlled to protect the privacy and confidentiality of participants in the Genomics England 100,000 Genomes Project and to comply with the consent given by participants for use of their healthcare and genomic data. Access to full data is permitted through the Research Network (https://www.genomicsengland.co.uk/research/academic/join-research-network), and by contacting the corresponding author upon reasonable request.

For TOPMed, a detailed description of the TOPMed participant consents and data access is provided in Box 130. TOPMed data used in this manuscript are available through dbGaP. The dbGaP accession numbers for all TOPMed studies referenced in this paper are listed in Extended Data Tables 23. A complete list of TOPMed genetic variants with summary level information used in this manuscript is available through the BRAVO variant browser (bravo.sph.umich.edu). The TOPMed imputation reference panel described in this manuscript can be used freely for imputation through the NHLBI BioData Catalyst at the TOPMed Imputation Server (https://imputation.biodatacatalyst.nhlbi.nih.gov/). DNA sequence and reference placement of assembled insertions are available in VCF format (without individual genotypes) on dbGaP under the TOPMed GSR accession phs001974.

For the 1000 Genomes Project, the WGS datasets are available from the European Nucleotide Archive under accessions PRJEB31736 (unrelated samples) and PRJEB36890 (related samples).

Research involving human participants, their data, or biological material

Policy information about studies with human participants or human data. See also policy information about sex, gender (identity/presentation), and sexual orientation and race, ethnicity and racism.

Reporting on sex and gender	Sex used in the paper matches with the genetically inferred sex.
Reporting on race, ethnicity, or other socially relevant groupings	The genetic ancestry used in the paper is based on the 1000 Genomes Project Consortium original work (https://www.nature.com/articles/nature15393), using a random forest model trained to predict five broad super-populations: African (AFR), Admixed American (AMR), East Asian (EAS), European (EUR), and South Asian (SAS) ancestries based on principal component analysis
Population characteristics	For each participant in the 100K GP, age was calculated based on the year of birth. Clinical data was entered by health-care professionals based on eligibility criteria and rare disease model (https://files.genomicsengland.co.uk/forms/Rare-Disease-Data-Model-Conditions-Phenotypes-and-Clinical-Tests-v1.9.0.pdf).<br>In the 100K GP 53.5% are female and 46.5% male, whereas in TOPMed 63.5% are female and 36.5% males. Median age was 50 in the 100K GP, and 62 in TopMed. The following populations were identified by genetic ancestry predictions in the 100K GP and TOPMed cohorts respectively: Africans (3.5%, 24%), Americans (1.8%, 10.5%), East Asians (0.9%, 2%), Europeans (86%, 63%), South Asians (7.5%, 0.7%).
Recruitment	For the 100K GP, participants were recruited by health-care professionals and researchers from 13 Genomic Medicine Centres in England, and were enrolled in the project if they or their guardian provided written consent for their samples and data to be used in research, including this study. Recruitment of TOPMed cohorts was performed at multiple sites within the US according to diverse inclusion and exclusion criteria, as described in the dbGAP entries for each cohort. We can provide more detailed information.
Ethics oversight	Genomics England has approval from the HRA Committee East of England – Cambridge South (REC Ref 14/EE/1112). For TOPMed, the study was approved by, and the procedures followed were in accordance with, the ethical standards of the Institutional Review Board of the Icahn School of Medicine under HS# 19-01376 and HS# 23-00469.

Note that full information on the approval of the study protocol must also be provided in the manuscript.

Field-specific reporting

Please select the one below that is the best fit for your research. If you are not sure, read the appropriate sections before making your selection.

☒ Life sciences ☐ Behavioural & social sciences ☐ Ecological, evolutionary & environmental sciences

For a reference copy of the document with all sections, see nature.com/documents/nr-reporting-summary-flat.pdf

Life sciences study design

All studies must disclose on these points even when the disclosure is negative.

Sample size	Any WGS data available across the 3 cohorts (100KGP, TopMed and 1KG) was included in the study if :

	-genomes sequenced following using PCR-free whole genome sequences AND mapped against Human GRCh38/hg38 assembly AND sequenced with a read-length 150bp.
Data exclusions	Genetically related genomes having up to third degree familiar relationship were excluded in all datasets. This is, only unrelated genomes were included in each cohort. Furthermore, In 100KGpand TopMed all genomes from individuals with a neurological disorder were excluded from this analysis (as these cohorts are medical sequencing studies that may have an over-representation of repeat expansions in people with a neurological disease)
Replication	Not applicable to this cross-sectional study
Randomization	Not applicable to this cross-sectional study
Blinding	Not applicable to this cross-sectional study

Reporting for specific materials, systems and methods

We require information from authors about some types of materials, experimental systems and methods used in many studies. Here, indicate whether each material, system or method listed is relevant to your study. If you are not sure if a list item applies to your research, read the appropriate section before selecting a response.

Materials & experimental systems

n/a	Involved in the study
☒	☐ Antibodies
☒	☐ Eukaryotic cell lines
☒	☐ Palaeontology and archaeology
☒	☐ Animals and other organisms
☐	☒ Clinical data
☒	☐ Dual use research of concern
☒	☐ Plants

Methods

n/a	Involved in the study
☒	☐ ChIP-seq
☒	☐ Flow cytometry
☒	☐ MRI-based neuroimaging

Clinical data

Policy information about clinical studies

All manuscripts should comply with the ICMJE guidelines for publication of clinical research and a completed CONSORT checklist must be included with all submissions.

Clinical trial registration	Not relevant as this study is not an interventional trial
Study protocol	Not relevant as this study is not an interventional trial
Data collection	Data analysis was carried out from April 2020 to April 2023.
Outcomes	Frequency of repeat expansion mutation and distribution across different populations
